# Impacts of Millipedes on Acari and Collembola Communities—A Microcosm Experiment

**DOI:** 10.3390/insects15060456

**Published:** 2024-06-18

**Authors:** Wenjin Chang, Peng Zhang, Jianwei Li, Nonillon M. Aspe, Jiahua Hao, Siyuan Lu, Zhuoma Wan, Donghui Wu

**Affiliations:** 1 State Environmental Protection Key Laboratory of Wetland Ecology and Vegetation Restoration, School of Environment, Northeast Normal University, Changchun 130117, China; changwj162@nenu.edu.cn (W.C.); lijw092@nenu.edu.cn (J.L.); haojh194@nenu.edu.cn (J.H.); lusy088@nenu.edu.cn (S.L.); wanzm984@nenu.edu.cn (Z.W.); 2 Key Laboratory of Wetland Ecology and Environment, Northeast Institute of Geography and Agroecology, Chinese Academy of Sciences, Changchun 130102, China; zhangpeng1@iga.ac.cn; 3 College of Marine and Allied Sciences, Mindanao State University at Naawan, Naawan 9023, Misamis Oriental, Philippines; nonillon_aspe@yahoo.com; 4 Key Laboratory of Vegetation Ecology, Ministry of Education, Northeast Normal University, Changchun 130024, China; 5 Jilin Provincial Key Laboratory of Animal Resource Conservation and Utilization, Northeast Normal University, Changchun 130117, China

**Keywords:** soil macrofauna, soil microarthropods, ecosystem engineers, community dynamics, biodiversity

## Abstract

**Simple Summary:**

Interactions among soil organisms are crucial for food webs and ecological functions. However, the effects of millipedes, which play a key role as decomposers in the soil, on soil microarthropods remain unclear. This study demonstrates that millipede activity in the soil decreased the abundance and diversity of Collembola in the soil while increasing their abundance and diversity in the litter layer. Consequently, this led to an increase in the abundance and diversity of Acari in the soil. The dominant species of both Acari and Collembola were most notably affected by the experiment. Millipede activity significantly altered the community structure of Acari and Collembola and strengthened the correlation between these two groups. These findings enhance our understanding of the ecological role of millipedes.

**Abstract:**

Ecosystem engineers influence the structure and function of soil food webs through non-trophic interactions. The activity of large soil animals, such as earthworms, has a significant impact on the soil microarthropod community. However, the influence of millipedes on soil microarthropod communities remains largely unknown. In this microcosm experiment, we examined the effects of adding, removing, and restricting millipede activity on Acari and Collembola communities in litter and soil by conducting two destructive sampling sessions on days 10 and 30, respectively. At the time of the first sampling event (10 d), Acari and Collembola abundance was shown to increase and the alpha diversity went higher in the treatments with millipedes. At the time of the second sampling event (30 d), millipedes significantly reduced the Collembola abundance and alpha diversity. The results were even more pronounced as the millipedes moved through the soil, which caused the collembolans to be more inclined to inhabit the litter, which in turn resulted in the increase in the abundance and diversity of Acari in the soil. The rapid growth of Collembola in the absence of millipedes significantly inhibited the abundance of Acari. The presence of millipedes altered the community structure of Acari and Collembola, leading to a stronger correlation between the two communities. Changes in these communities were driven by the dominant taxa of Acari and Collembola. These findings suggest that millipedes, as key ecosystem engineers, have varying impacts on different soil microarthropods. This study enhances our understanding of biological interactions and offers a theoretical foundation for soil biodiversity conservation.

## 1. Introduction

Interactions between organisms are major determinants of biodiversity, species distribution, and abundance [1,2,3], profoundly affecting ecosystem stability [4]. Soil is among the most species-rich habitats on Earth [5], and the animals living within it play a crucial role in nutrient cycling, material utilization, and assessing soil health [6,7]. Most current studies focus on aboveground–belowground interactions, animal–plant relationships, and microbial interactions [8,9,10]. However, the mechanisms of interactions among soil organisms and their impacts on ecosystem processes are still not well understood. Soil invertebrates play crucial roles in key ecological interactions [11], and their function as ecosystem engineers significantly enhances species richness [12].

Forty-five percent (45%) of interactions in the soil are generated by burrowing ecosystem engineers [8]. Larger invertebrates influence the diversity of smaller organisms by facilitating their dispersal and modifying soil habitats [13,14]. Millipedes, considered important soil ecosystem engineers, are abundant, diverse, and widely distributed [11,15,16]. They impact soil fauna habitats to varying degrees [17] and enhance soil aeration by creating a more porous soil structure through the accumulation of excavated soil or manure [18,19,20]. Some species have been shown to have significant effects on microbial biomass, enzymatic activity, and soil aggregates [21,22,23,24]. Their feces has been used as an indicator of organic matter turnover, altering organic matter and C and N stabilization [25,26]. Compared to earthworms, millipedes have a greater ability to break down litter and alter the state of the litter, thereby altering food resources [18].

Acari and Collembola, key constituents of soil microarthropods, are among the most abundant and diverse soil fauna [27,28]. Serving as indicator organisms of soil quality [29,30], they occupy various soil and litter layers [31]. These organisms rely on soil pore space and food resources such as litter and microorganisms for survival [32,33,34,35]. Acari and Collembola constitute a significant portion of decomposer communities, with their dominant species being particularly sensitive to environmental changes [36,37,38]. Larger ecosystem engineers, like earthworms, affect Acari and Collembola differently by altering soil structure and food resources, thereby changing community composition and relative abundance [32,39,40].

Research on the interactions between millipedes and other organisms has primarily focused on their relationships with other soil macrofauna, such as earthworms, and how these associations influence litter decomposition, alter the soil environment, and regulate microorganisms [41,42]. Currently, there is very limited information on the effects of millipedes on soil microarthropod communities. Therefore, further experiments are needed to explore the role of millipedes on microarthropods [43]. In this work, we investigated the effects of millipedes on Acari and Collembola communities in the soil. Our hypotheses revolve around three key points: (a) millipedes differentially affect the structure and composition of Acari and Collembola communities while altering their distribution in soil and litter; (b) the presence and activity of millipedes contribute to alterations in the relationship between Acari and Collembola; and (c) the most pronounced changes within the Acari and Collembola communities will be observed through their dominant species.

## 2. Materials and Methods

### 2.1. Study Area

The materials used in the experiments were obtained from the Changchun Agricultural Positioning Experiment Station, Northeast Institute of Geography and Agroecology, Chinese Academy of Sciences (44°59′ N, 125°23′ E), in Changchun City, Jilin Province, China. The area has a temperate continental climate with an average annual temperature of 4.6 °C–6.4 °C and average annual rainfall of 614 mm. The type of soil is black (Typic Hapludoll, USDA, 1993) with clay loam texture. *Orthomorphella pekuensi* (Karsch, 1881) is a millipede widely distributed in China [44] and is the dominant large soil animal in the study site, measuring about 20 to 35 mm in length. Like most millipedes, *O. pekuensi* is a scavenging species that feeds primarily on plant litter or organic residues in various stages of decomposition. The litter of *Populus tomentosa* Carr was selected as the study material as it is the dominant tree species in the study site. It is also one of the main food sources for millipedes and soil microarthropods. The experiment was conducted in mid-August, when the study site was in the highest number and it was the most active time for the millipedes.

### 2.2. Microcosmic Experimental Design

**Figure 1:**
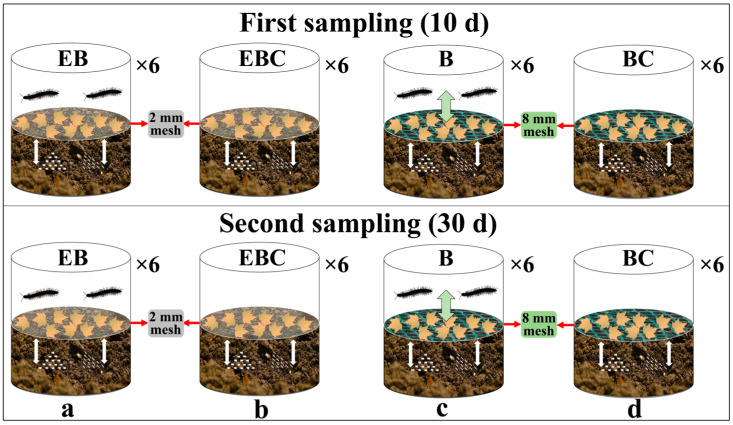
Schematic diagram of the four microcosms used in the experiment. (**a**) EB: exclusion burrowing (2 mm mesh and millipedes); (**b**) EBC: control for exclusion burrowing (2 mm mesh); (**c**) B: burrowing (8 mm mesh and millipedes); (**d**) BC: control for burrowing (8 mm mesh). Six replicates of each treatment. Sampled in two waves (10 and 30 days). The white dots represent Acari and Collembola. White double arrows represent Acari and Collembola that can move between the litter layer and the soil layer. Green double arrows represent millipedes that can move between the litter layer and the soil layer.

The experiment utilized four microcosmic treatments as follows: (a) Millipedes were introduced to the litter layer, with a 2 mm mesh barrier placed between the litter layer and the soil layer to prevent millipedes from burrowing into the soil while allowing the activities of Acari and Collembola. This treatment is abbreviated as exclusion burrowing (EB). (b) A 2 mm mesh barrier was added in the same manner as (a), but without millipedes, serving as a control for exclusion burrowing (EBC). (c) Millipedes were added to the litter layer, and to avoid interference from the mesh, an 8 mm mesh was added to both the litter and soil layers, allowing millipedes to burrow into the soil. This treatment is abbreviated as burrowing (B). (d) An 8 mm mesh was added without millipedes, serving as a control for burrowing (BC) (see Figure 1). The 2 mm mesh permitted the movement of Acari and Collembola between the soil and litter layers while preventing millipedes from passing through the mesh [45]. Conversely, the 8 mm mesh did not impede the movement of millipedes along with Acari and Collembola. The experiment spanned a duration of 30 days, during which, two destructive sampling sessions were conducted on days 10 and 30, taking into account the impact of millipedes on feces production and soil structure alteration within this timeframe. Each microcosm treatment was replicated six times, resulting in a total of 48 microcosms for the experiment. Each microcosm comprised a cylindrical PVC plastic cup measuring 130 mm in height, with a bottom diameter of 83 mm and a mouth diameter of 95 mm. Small 0.1 mm filters were fitted onto the lids of the plastic cups to prevent soil animals from escaping while maintaining air permeability (see Figure 1).

### 2.3. Experimental Material Collection and Pre-Processing

#### 2.3.1. Millipede Collection and Pre-Processing

All millipedes used in the experiment were collected manually from the Positioning Experimental Station. Samples were taken two days after the rain, when the soil had the optimum moisture content (between 17% and 28%). Millipedes and soil microarthropods were more active at this time. The millipedes were incubated in four boxes before being used for the experiment. The rearing conditions were as follows: temperature of 20 °C, photoperiod 12 h/12 h, and air humidity of 40% (referring to the same conditions as the sampling site). The microcosms were misted with water on a weekly basis to keep the millipedes in a moist environment. The millipedes were cultured for one month using soil (0–10 cm) and aboveground litter (moderately broken) from the collection site as the medium to acclimatize them to the laboratory culture environment. The larger adult millipedes were selected from the pre-cultured millipedes prior to the start of the experiment (body width greater than 2 mm and less than 8 mm). These millipedes were starved for 48 h to void their intestine.

#### 2.3.2. Soil Collection and Pre-Processing

A square sample plot measuring 20 × 20 m was designated at the millipede collection site. Within this plot, five points were selected: one at the center and four at the corners, each measuring 1 × 1 m. From these points, soil samples from the 0–5 cm depth were collected. The soil obtained from the five points was combined and manually sorted to remove larger roots, stones, and plant debris, as well as soil macrofauna visible to the naked eye [46]. These unfiltered soils retained their natural microarthropod communities. Prior to being introduced into the experiment, the soil was homogenized to ensure consistency in the Acari and Collembola communities and population numbers across each microcosm [47].

#### 2.3.3. Litter Collection and Pre-Processing

Litter at the site was collected at the same time soil samples were collected. Intact leaves were chosen and then cut into pieces approximately 0.8–2 cm^2^ in size [41]. To remove microarthropods and insect eggs from the litter while preserving the microbial community as effectively as possible, the leaves were dried in an oven at 70 °C for 24 h [48].

#### 2.3.4. Assembly of Microcosmic Experimental Setup

An average weight of 160 g of moist soil with its natural microarthropod community was introduced into each microcosm. The initial moisture content of the soil in each experimental unit was measured. Distilled water was added to reach 20% moisture content. The dried leaves were mixed and weighed, and 2 g of litter was added to each microcosm. Meanwhile, 2 mm and 8 mm mesh were inserted, respectively, between the litter and the soil to serve as barriers for the movement of soil fauna. The leaves were lightly sprayed with water to maintain moisture. Treatment microcosms were placed in an incubator at 20 °C for 48 h until the leaves were completely moist [43,49] (Figure 1).

### 2.4. Cultivation and Sample Collection for Microcosm Experiments

Once the microcosm was fully assembled, millipedes of similar weight and length were introduced into the litter layer of the microcosm after they were starved. Two millipedes were added to each microcosm (consistent with the density of millipedes at the collection site). The survival rate of millipedes was assessed daily. The millipedes were kept in a moist environment by lightly spraying water every 2–3 days. If a millipede died before the end of the experiment, it was removed and replaced with a millipede of similar size and weight. The microcosm was maintained in an incubator subjected to constant temperature and soil moisture (20 °C and 20%, respectively) for 30 days. The activity and consumption of food resources by millipedes within this period was sufficient enough to have an impact on the soil [43].

The litter in each microcosm was carefully pinched out with tweezers, and then, all the soil in each microcosm was poured out. The litter and soil were placed on separate labeled sieves using the modified Tullgren funnel method. After 10 days, samples were collected and stored in 95% ethanol at −20 °C and were prepared for identification (with the first sampling event randomly selecting 24 microcosms). All Acari and collembolans were separated and counted using a stereomicroscope. The adults were identified at the species level [50,51,52,53].

### 2.5. Statistical Analysis

The data were processed and analyzed using R 4.1.1. Analysis of variance (ANOVA) was used to compare the abundance of Acari and Collembola and community alpha diversity between different treatments in the litter and soil layers. If the data did not follow a normal distribution, the Kruskal–Wallis test was used. In order to compare the abundance and community alpha diversity of Acari and Collembola between litter layer and soil layer under the same treatment, a T-test was used, and a non-parametric test was used to test the data with great deviation. Principal Coordinate Analysis (PCoA) was utilized to elucidate the distribution patterns of Acari and Collembola under different treatments. Furthermore, differences in community composition under different treatments were examined through the permutational multivariate analysis of variance (PERMANOVA). These analyses were conducted using the “vegan” package [54]. In order to compare the relationship between different species of Acari and Collembola, correlation heatmaps were constructed using Pearson correlation coefficients. The creation of these heatmaps relied on the implementation of the “pheatmap” package [55]. The classification of dominant, common, and rare taxa was based on the relative abundance table of Acari and Collembola, utilizing the following criteria: taxa with an individual count exceeding 10% of the total number were classified as dominant, taxa with a count between 1% and 10% were classified as common, and taxa below 1% were classified as rare [56]. Significance levels were established as follows: correlations and differences were deemed significant at *p* < 0.05, particularly significant at *p* < 0.01, and highly significant at *p* < 0.001.

## 3. Results

### 3.1. Abundance and Composition of Acari and Collembola Communities

**Figure 2:**
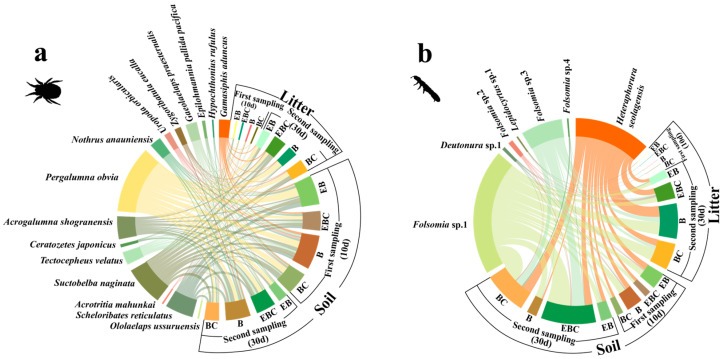
Relative abundance of (**a**) Acari and (**b**) Collembola species in different treatments. Different colors represent different species with different treatments.

In this experiment, a total of 2195 Acari and Collembola individuals were collected. Among these, there were 15 species of Acari from 15 genera and 13 families and 7 species of Collembola from 4 genera and 4 families. In the initial sampling event, no significant impact on the abundance of Acari and Collembola in the litter was observed in the EBC and BC treatments. However, the presence of millipedes (EB and B) significantly increased the abundance of Acari (*p* < 0.001) and Collembola (*p* < 0.01) in the soil (refer to Figure 2 and Table S1). In the subsequent sampling event, a notable increase was observed in the abundance of Acari in the soil in treatment B (*p* < 0.05), while there was a decrease in the abundance of both Acari and Collembola in the EB treatment (*p* < 0.001) (Figure 2, Table S2).

The same species were clearly growing more in the soil than in the litter at the time of the first sampling event (Figure 2, Table S3). At the time of the second sampling event, there was a significant increase in Collembola abundance in the litter of the B treatment (*p* < 0.001), while there was a significant decrease in Collembola abundance in the litter layer vs. the soil layer in the BE treatment (*p* < 0.001) (Figure 2, Table S4). There was significantly higher Acari and Collembola abundance in the soil than in the litter in the control groups (EBC and BC) (*p* < 0.05) (Table S4).

The dominant Acari taxa in the soil at the time of the first sampling event were *Scheloribates reticulatus* of Scheloribatidae, *Suctobelba naginata* of Suctobelbidae, and *Pergalumna obvia* of Galumnidae, which accounted for 49.33–71.17% of the total Acari species. *Heteraphorura seolagensis* and *Folsomia* sp.1 accounted for 88.73–100% of the overall Collembola population. The addition of millipedes increased the common taxa of Collembola with rare taxa of Acari (Figures S1 and S2). The dominant Acari taxa in the second sampling event of litter and soil were *Scheloribates reticulatus*, *Suctobelba naginata*, and *Pergalumna obvia* and *Acrogalumna shogranensis* of Galumnidae, which accounted for 52.12–73.34% of the total Acari species. Meanwhile, *Heteraphorura seolagensis*, *Folsomia* sp.1, and *Folsomia* sp.3 accounted for 86.49%–100% of the total Collembola species. The addition of millipedes reduced the dominant taxa of Acari in litter and soil (Figures S1 and S2).

### 3.2. Changes in Acari and Collembola Communities in Litter and Soil under Different Treatments

**Figure 3:**
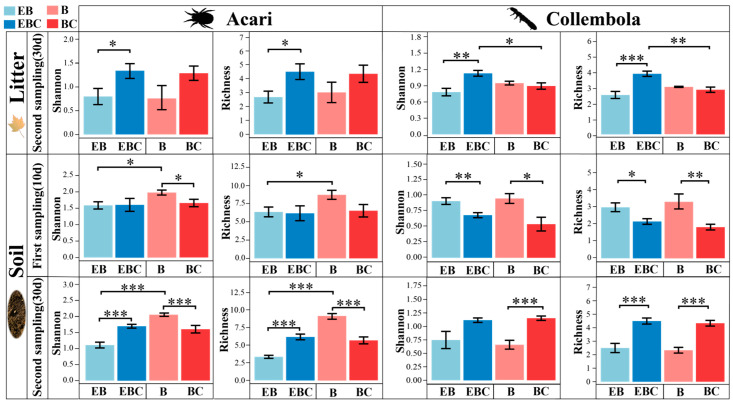
Differences in the richness and Shannon–Wiener indexes of Acari and Collembola in litter layer and soil layer between different treatments. (* *p* < 0.05; ** *p* < 0.01; *** *p* < 0.001).

The number of Acari and Collembola collected in litter layer during the first sampling event was very small. At the time of the first sampling event of the soil, the diversity of Collembola increased in the EB and B treatments (*p* < 0.01), while the diversity of Acari significantly increased in the B treatment (*p* < 0.05) (Figure 3). In the second sampling event, the diversity of Acari and Collembola in the litter and soil significantly decreased in the EB treatment (*p* < 0.001 and *p* < 0.001, respectively). Compared with EB treatment, B treatment increased the diversity of Acari in soil (*p* < 0.001) and decreased the diversity of Collembola in soil (*p* < 0.001) but had no significant effect on Acari and Collembola in litter (Figure 3).

**Figure 4:**
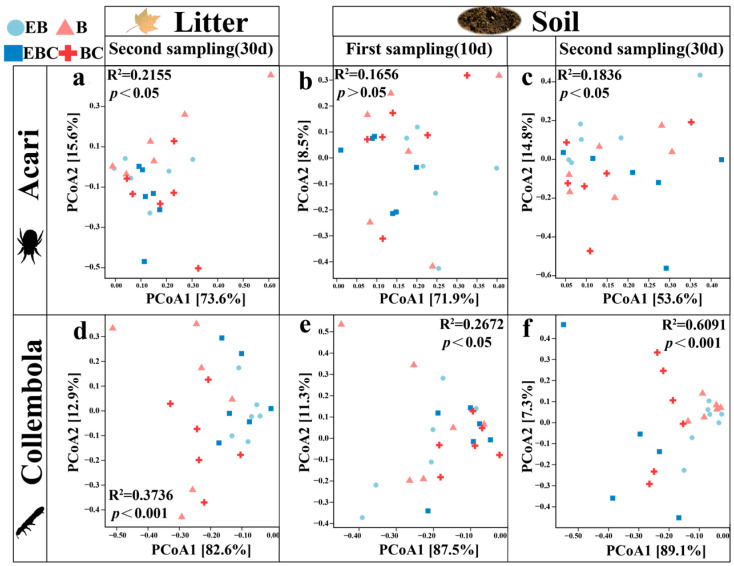
Differences in community structure of Acari and Collembola in litter layer and soil layer under different treatments. (Different colors and shapes represent different treatments.)

The number of Acari and Collembola colonizing the litter for the first time was small and insufficient to analyze the community structure. However, the change in the community structure of Collembola in the soil was more pronounced (R^2^ = 0.2672; *p* < 0.05) (Figure 4e). In the second sampling event, the presence of millipedes (EB and B) caused a significant change in the Acari and Collembola communities and a more pronounced change in the Collembola community structure (R^2^ = 0.6091; *p* < 0.001) (Figure 4). The EB treatments had a greater effect on Acari in the soil, and the B treatments had a greater effect on the Collembola community structure in the soil (Figure 4).

**Figure 5:**
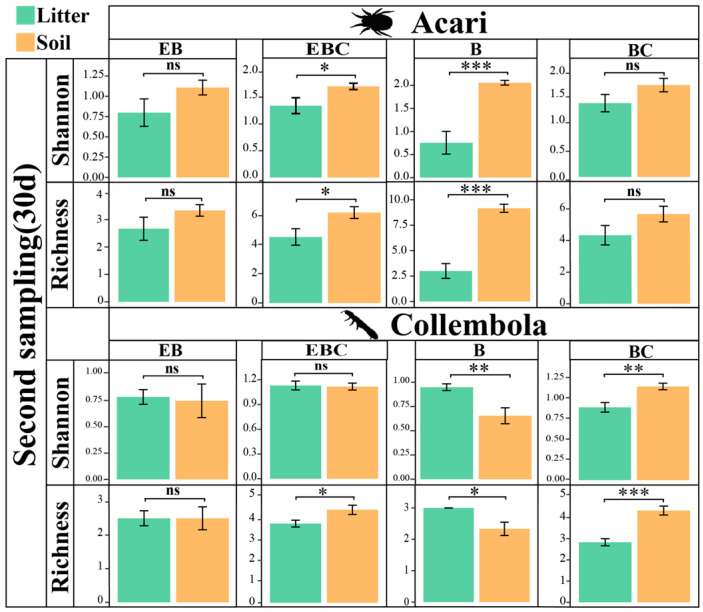
The richness and Shannon–Wiener indexes of Acari and Collembola were different between litter layers and soil layers under different treatments at the time of the second sampling event. (“ns” represents no significant difference. * *p* < 0.05; ** *p* < 0.01; *** *p* < 0.001).

At the time of the second sampling event, the diversity of Acari in the soil and Collembola in the litter significantly increased in the B treatment (*p* < 0.001 and *p* < 0.01, respectively). Exclusion burrowing (EB) significantly reduced the diversity of Acari and Collembola in the litter (*p* < 0.05) (Figure 5).

**Figure 6:**
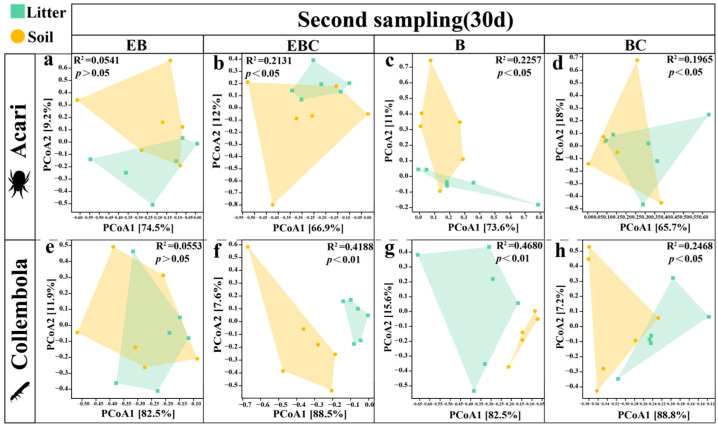
Differences in Acari and Collembola community structure in the litter and soil in different treatments at the time of the second sampling event (Subfigures (**a**–**d**) show the differences in community structure between the litter and soil layers of the Acari community under the four different treatments. Subfigures (**e**–**h**) show the differences in community structure between the litter and soil layers of the Collembola community under the four different treatments.).

Comparing the litter and soil layers, the community structure of Acari and Collembola was significantly different in the control groups (EBC and BC) and burrowing (B) treatments (*p* < 0.05) (Figure 6). The EB treatments did not have a significant effect on Acari and Collembola community structures (Figure 6a,e).

### 3.3. Relationship between Acari and Collembola

In the first sampling event, the dominant Acari and Collembola species in the litter and soil showed a significant positive correlation (*p* < 0.05) (Figure S3), which were made more pronounced with the addition of millipedes (EB and B) (*p* < 0.001) (Figure S3). In the second sampling event, burrowing (B) intensified the positive correlation (*p* < 0.05) (Figure S3).

**Figure 7:**
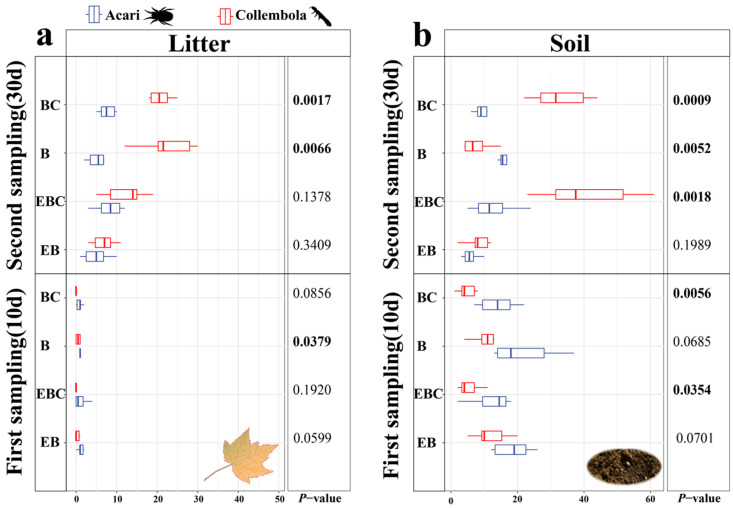
Subfigure (**a**) show the total number of Acari compared to the total number of Collembola at the two sampling times in the litter layer. Subfigure (**b**) show the total number of Acari compared to the total number of Collembola at the two sampling times in the soil layer (Bolded *p*-values are significant differences).

With regard to the overall abundance of Acari and Collembola, significant changes were observed in the control groups (EBC and BC) (Figure 7). Acari abundance was significantly higher than Collembola abundance in the soil in the B treatment in the second sampling event (*p* < 0.01) (Figure 7b), while Collembola abundance was significantly higher than Acari in the litter layer (*p* < 0.01) (Figure 7a).

## 4. Discussion

### 4.1. Effect of Mechanical Disturbance of Millipedes on Acari and Collembola

The presence and burrowing activities of millipedes had a strong influence on Acari and Collembola in the soil and litter (Figures S1 and S2; and Tables S3 and S4), supporting Hypothesis a. Millipedes burrowed in the soil (B), creating numerous visible pores and a softer texture at the end of the experiment, while the large number of different shapes and sizes of feces produced by millipedes changed the soil structure (Figure S5a,b). Millipedes affect small soil animals and microorganisms by altering the physical environment and creating microhabitats [14]. Soil quality is heavily influenced by the activity of soil macrofauna. Similar to other ecosystem engineers like earthworms and ants, millipedes enhance soil pore space by their burrowing activities, consequently improving soil permeability [20,57]. Various species of Collembola are adapted to different soil depths and microhabitat conditions [58]. Studies have suggested that the increase in Folsomia spp. populations may be linked to changes in soil porosity induced by earthworms [59,60]. Oribatid mites, a type of Acari, primarily feed on detritus and fungi, typically inhabiting organic and litter layers [61]. Although millipedes mostly dwell near the soil surface or beneath litter, their digging behavior, akin to earthworms, can transport more food resources deeper into the soil [62]. This behavior facilitates soil-dwelling microarthropods’ access to food, aiding in their survival. When facing millipedes in the soil, similar to the effects of earthworms on soil structure, Collembola find it easier to access the soil and evade predators [63]. Collembola, being more mobile than Acari, tend to survive better in the litter layer when predatory Acari populations increase in the soil. Additionally, frequent mechanical disturbance of the soil diminishes the density and abundance of Collembola [64]. This dynamic results in a higher diversity and abundance of Acari in the soil compared to the litter layer (refer to Figure 2 and Figure 4; and Tables S3 and S4). It has been proposed that Acari may exhibit a stronger symbiotic relationship with millipedes [15]. However, different millipede species, with varying body sizes and behaviors, may exert differing impacts on soil microarthropods, necessitating further research on the ecological implications of different soil fauna.

### 4.2. The Relationship between Acari and Collembola

The presence and activity of millipedes changed the relationship between Acari and Collembola (Hypothesis b) and had a positive effect on the population of some species of both groups (Figures S3 and S4). Acari abundance decreased and community structure was disturbed during the second sampling event, but the diversity was not negatively affected. Collembola reduced in abundance and diversity when confronted with millipedes (Figure 2 and Figure 3 and Tables S1 and S2). Mutualistic symbiosis may be the most variable species relationship among multiple interactions relative to others [65]. Positive effects at the time of the first sampling may be due to the formation of microhabitats, which provide more space and resources for their survival. Reciprocal interactions, as opposed to competitive interactions, depend largely on abiotic factors, and they become more important under extreme conditions according to the stress gradient hypothesis (SGH) [66]. An example is the instability of microbial communities in response to environmental stresses [67]. For Acari and Collembola to respond differently, it is possible that the stress due to environmental change leads to differences between communities. Oribatid mites have long been recognized as k-selective organisms with slow metabolisms, low fecundity, and little ability to respond quickly to environmental changes [68,69]. Consequently, their populations decline when their favorable habitat is disturbed. The low reproductive rates of most oribatid species may be slow to recover from these disturbances [70]. In contrast, several predatory mites, such as the Mesostigmata, were essentially unchanged in the experiments (Tables S1 and S2), probably because they are usually predators of other microarthropods, larvae, and nematodes [71,72]. In contrast to oribatids, mite-predatory microarthropods in a mesocosm exert predatory pressure on soil microarthropods, while their resistance to environmental change may be high due to a wider range of feeding options [73,74]. Among the soil fauna, Collembola are considered as a rapid response group (r-strategist) to environmental changes. They are more prolific and react more quickly in the face of disturbances [64,75]. Therefore, there was a sharp increase in the number of Collembola species during the second sampling event, and this greater density reduced the space available for Acari, leading to a decrease in their abundance. When the Collembola community showed a decreasing trend, the competition for food resources may have been reduced, so the abundance and diversity of the Acari community increased significantly.

### 4.3. Main Mechanisms Driving the Relationship of Millipedes with Acari and Collembola

Overall, the presence of millipedes in this experiment had a significant impact on the abundance, diversity, and community structure of Acari and Collembola (Figure 2, Figure 3 and Figure 4, and Tables S1 and S2). The presence of soil macrofauna has a strong influence on the soil microarthropods by altering the soil structure, food resources, and their interactions such as predation and competition [40,76]. Millipedes not only provide litter resources directly to soil microorganisms, but also alter the physical state of the litter and soil [21]. Millipedes differ from earthworms in that they have more developed mouthparts, which break up the litter to produce finer debris during the feeding process, increasing the range of food resources available to the microarthropods by providing easier access to them [77]. The genus *Folsomia* has a small percentage of fungi in its gut [34], and fungi are not a major food resource for this Collembola genus [78]. Members of this genus are thought to feed on litter and detritus [79,80]. The distribution of Acari is somewhat related to resource constraints [33]. Some Acari of the genus *Scheloribates* are categorized as pan-phytophagous mites, which have numerous distributions in grassland soils [81], and plant litter is an important food source for this species [82]. Certain Acari species, such as *Tectocepheus*, exhibit broad dietary preferences, consuming various resources from lichens to fungi [83]. However, there were no notable changes in abundance observed across the treatments (Tables S1 and S2). *Pergalumna* spp. of the Galumnidae family, under the isotope test, were shown to feed on a wide range of resources, including live nematodes and collembolans [84]. They have a wider range of food choices and have been observed to be more stable to millipede disturbance, and thus, were not significantly affected across the treatments.

It was observed in the experiment that a large amount of millipede feces accumulated on the soil surface or in the litter layer (Figure S5). The persistence of millipede feces resulted in a longer accumulation of soil carbon [21]. Their crushing of the litter for partial digestion and repackaging into feces increased the instability of the organic matter [25]. The presence of this feces changes the composition of the microbial community in the soil [42,43], which in turn changes the allocation of its food resources, leading to changes in the Acari and Collembola communities. Acari and Collembola have a wide range of dietary habits [34,85], and the presence of leaf litter and the abundance of fungi provide abundant food resources for Acari and Collembola that feed on detritus and fungi [86]. The mite species *Acrogalumna shogranensis*, which was dominant throughout the experiments, had a strong susceptibility to millipede activity (Figure 2; and Tables S3 and S4). Most *Acrogalumna* species feed on fungi [87]. Changes in the dominant species of the Acari and Collembola communities were most pronounced when millipedes were present in the different treatments (Figure 2 and Figures S1, S3 and S4). The abovementioned changes in dominant species also proved to support Hypothesis c. As Acari and Collembola are the two dominant microarthropod groups, their abundance and diversity drive the overall microarthropod community [86]. The dominant species of these groups play an important role in maintaining biodiversity in the soil and in the stabilization of the ecosystem through the decomposition and recycling of materials, and the presence of millipedes may increase food resources and survival space for the microarthropod groups.

## 5. Conclusions

This study showed that millipedes had different effects on different species of Acari and Collembola. In the short term, millipedes significantly increased the abundance and diversity of soil Acari and Collembola, but the rapid growth of Collembola over time suppressed the Acari community. Millipede burrowing predisposes collembolans to colonize the litter layer, resulting in an increase in soil Acari abundance and diversity. Millipedes altered the structure and composition of the Acari and Collembola communities and intervened in their interactions. These changes were driven by the dominant species in the Acari and Collembola communities. These results provide support for the effects of soil macrofauna on soil microarthropod communities to better understand the interactions between soil organisms and the effects on soil biodiversity. Further studies are needed to investigate the main drivers of the relationship between millipedes and soil microarthropods.

## Data Availability

Data will be made available on request.

## Supplementary Materials

The following supporting information can be downloaded at https://www.mdpi.com/article/10.3390/insects15060456/s1: Figure S1. Stacked plots with percentage abundance of (a) Acari and (b) Collembola species in different treatments under two sampling sessions. Heatmap of correlation between different species under different treatments at time of first sampling (c) and second sampling event (d); Figure S2. (a) Venn diagram of species in the litter and soil under two sampling sessions. (b) Bubble diagram of the distribution of different species under different treatments; Figure S3. Heatmap of correlations between different species of acari and collembola under different treatments in the litter and soil. (Red is positive correlation and blue is negative correlation; the darker the color and the more complete the pie shape, the stronger the correlation. * *p* < 0.05; ** *p* < 0.01; *** *p* < 0.001); Figure S4. Heatmap of correlation between Acari and Collembola in different treatments. (Red is positive correlation and blue is negative correlation; the darker the color and the more complete the pie shape, the stronger the correlation.); Figure S5. a. The pores produced by the millipede activity in the soil, and it can also be observed that there is more water in the pores; b. the soil without millipede treatment; the soil as a whole is highly compacted without large pores; c. the feces left by the millipede in the litter layer and the breaking of the litter edge; d. the large amount of feces left by the millipede in the soil; all the round pie-shaped middle depressions in the figure are the feces produced by the millipede feces; Table S1. Differences in Acari and Collembola abundance between treatments in the litter and soil at the time of the first sampling event; Table S2. Differences in Acari and Collembola abundance between treatments in the litter and soil at the time of the second sampling event; Table S3. Differences in Acari and Collembola abundance in contrasting soils in the litter under different treatments at the time of the first sampling event; Table S4. Differences in Acari and Collembola abundance in contrasting soil in the litter under different treatments at the time of the second sampling event.

## References

[1] Wilson W.G., Lundberg P., Vázquez D.P., Shurin J.B., Smith M.D., Langford W., Gross K.L., Mittelbach G.G. (2003). Biodiversity and species interactions: Extending Lotka–Volterra community theory. Ecol. Lett..

[2] Ricklefs R.E. (2004). A comprehensive framework for global patterns in biodiversity. Ecol. Lett..

[3] Brophy C., Dooley Á., Kirwan L., Finn J.A., McDonnell J., Bell T., Cadotte M.W., Connolly J. (2017). Biodiversity and ecosystem function: Making sense of numerous species interactions in multi-species communities. Ecology.

[4] Gorter F.A., Manhart M., Ackermann M. (2020). Understanding the evolution of interspecies interactions in microbial communities. Philos. T. R. Soc. B..

[5] Bardgett R.D., van der Putten W.H. (2014). Belowground biodiversity and ecosystem functioning. Nature.

[6] Swift M.J., Andrén O., Brussaard L., Briones M., Couteaux M.-M., Ekschmitt K., Kjoller A., Loiseau P., Smith P. (1998). Global change, soil biodiversity, and nitrogen cycling in terrestrial ecosystems: Three case studies. Global Chang. Biol..

[7] Bagyaraj D.J., Nethravathi C.J., Nitin K.S. (2016). Soil Biodiversity and Arthropods: Role in Soil Fertility. Economic and Ecological Significance of Arthropods in Diversified Ecosystems.

[8] Wurst S., Sonnemann I., Zaller J.G., Ohgushi T., Wurst S., Johnson S.N. (2018). Soil macro-invertebrates-their impact on plants and associated aboveground communities in temperate regions. Aboveground-Belowground Community Ecology.

[9] Bennett J.A., Klironomos J. (2019). Mechanisms of plant–soil feedback: Interactions among biotic and abiotic drivers. New Phytol..

[10] Brambilla M., Scridel D., Bazzi G., Ilahiane L., Iemma A., Pedrini P., Bassi E., Bionda R., Marchesi L., Genero F. (2020). Species interactions and climate change: How the disruption of species co-occurrence will impact on an avian forest guild. Global Chang. Biol..

[11] Auclerc A., Beaumelle L., Barantal S., Chauvat M., Cortet J., De Almeida T., Dulaurent A.-M., Dutoit T., Joimel S., Séré G. (2022). Fostering the use of soil invertebrate traits to restore ecosystem functioning. Geoderma.

[12] Romero G.Q., Gonçalves-Souza T., Vieira C., Koricheva J. (2015). Ecosystem engineering effects on species diversity across ecosystems: A meta-analysis. Biol. Rev..

[13] Brown G.G. (1995). How Do earthworms affect microfloral and faunal community diversity?. Plant Soil.

[14] Wardle D.A. (2006). The influence of biotic interactions on soil biodiversity. Ecol. Lett..

[15] Eisenhauer N., Partsch S., Parkinson D., Scheu S. (2007). Invasion of a deciduous forest by earthworms: Changes in soil chemistry, microflora, microarthropods and vegetation. Soil Biol. Biochem..

[16] Seeber J., Scheu S., Meyer E. (2006). Effects of macro-decomposers on litter decomposition and soil properties in alpine pastureland: A mesocosm experiment. Appl. Soil Ecol..

[17] Maran A.M., Pelini S.L. (2016). Predator contributions to belowground responses to warming. Ecosphere.

[18] Snyder B.A., Hendrix P.F. (2008). Current and potential roles of soil macroinvertebrates (Earthworms, Millipedes, and Isopods) in ecological restoration. Restor. Ecol..

[19] Lavelle P., Spain A., Blouin M., Brown G., Decaëns T., Grimaldi M., Jiménez J.J., McKey D., Mathieu J., Velasquez E. (2016). Ecosystem engineers in a self-organized soil: A review of concepts and future research questions. Soil Sci..

[20] Mele G., Buscemi G., Gargiulo L., Terribile F. (2021). Soil burrow characterization by 3D image analysis: Prediction of macroinvertebrate groups from biopore size distribution parameters. Geoderma.

[21] Toyota A., Kaneko N., Ito M.T. (2006). Soil ecosystem engineering by the train millipede *Parafontaria laminata* in a Japanese larch forest. Soil Biol. Biochem..

[22] Fujimaki R., Sato Y., Okai N., Kaneko N. (2010). The train millipede (*Parafontaria laminata*) mediates soil aggregation and N dynamics in a Japanese larch forest. Geoderma.

[23] Silva V.M.d., Antoniolli Z.I., Jacques R.J.S., Ott R., da S. (2017). Rodrigues, P.E.; Andrade, F.V.; Passos, R.R.; Mendonça, E. de S. Influence of the tropical millipede, *Glyphiulus granulatus* (Gervais, 1847), on aggregation, enzymatic activity, and phosphorus fractions in the soil. Geoderma.

[24] Bray N., Kao-Kniffin J., Frey S.D., Fahey T., Wickings K. (2019). Soil macroinvertebrate presence alters microbial community composition and activity in the rhizosphere. Front. Microbiol..

[25] Joly F.-X., Coq S., Coulis M., David J.-F., Hättenschwiler S., Mueller C.W., Prater I., Subke J.-A. (2020). Detritivore conversion of litter into faeces accelerates organic matter turnover. Commun. Biol..

[26] Coq S., Ganault P., Le Mer G., Nahmani J., Capowiez Y., Dignac M.-F., Rumpel C., Joly F.-X. (2022). Faeces traits as unifying predictors of detritivore effects on organic matter turnover. Geoderma.

[27] Petersen H., Luxton M. (1982). A comparative analysis of soil fauna populations and their role in decomposition processes. Oikos.

[28] Potapov A.M., Guerra C.A., van den Hoogen J., Babenko A., Bellini B.C., Berg M.P., Chown S.L., Deharveng L., Kováč Ľ., Kuznetsova N.A. (2023). Globally invariant metabolism but density-diversity mismatch in springtails. Nat. Commun..

[29] Santorufo L., Van Gestel C.A.M., Rocco A., Maisto G. (2012). Soil invertebrates as bioindicators of urban soil quality. Environ. Pollut..

[30] Culliney T.W. (2013). Role of arthropods in maintaining soil fertility. Agriculture.

[31] Potapov A.A., Semenina E.E., Korotkevich A.Y., Kuznetsova N.A., Tiunov A.V. (2016). Connecting taxonomy and ecology: Trophic niches of collembolans as related to raxonomic identity and life forms. Soil Biol. Biochem..

[32] Migge-Kleian S., McLean M.A., Maerz J.C., Heneghan L. (2006). The influence of invasive earthworms on indigenous fauna in ecosystems previously uninhabited by earthworms. Biol. Invasions.

[33] Mumladze L., Murvanidze M., Maraun M., Salakaia M. (2015). Oribatid mite communities along an elevational gradient in Sairme gorge (Caucasus). Exp. Appl. Acarol..

[34] LeFait A., Gailey J., Kernaghan G. (2019). Fungal species selection during ectomycorrhizal grazing by Collembola. Symbiosis.

[35] Lagendijk D.D.G., Cueva-Arias D., Van Oosten A.R., Berg M.P. (2022). Impact of three co-occurring physical ecosystem engineers on soil Collembola communities. Oecologia.

[36] Maraun M., Martens H., Migge S., Theenhaus A., Scheu S. (2003). Adding to ‘the enigma of soil animal diversity’: Fungal feeders and saprophagous soil invertebrates prefer similar food substrates. Eur. J. Soil Biol..

[37] Susanti W.I., Bartels T., Krashevska V., Widyastuti R., Deharveng L., Scheu S., Potapov A. (2021). Conversion of rainforest into oil palm and rubber plantations affects the functional composition of litter and soil Collembola. Ecol. Evol..

[38] Gergócs V., Flórián N., Tóth Z., Szili-Kovács T., Mucsi M., Dombos M. (2022). Crop species and year affect soil-dwelling Collembola and Acari more strongly than fertilisation regime in an arable field. Appl. Soil Ecol..

[39] McLean M.A., Parkinson D. (1998). Impacts of the epigeic earthworm *Dendrobaena octaedra* on oribatid mite community diversity and microarthropod abundances in pine forest floor: A mesocosm study. Appl. Soil Ecol..

[40] Eisenhauer N. (2010). The action of an animal ecosystem engineer: Identification of the main mechanisms of earthworm impacts on soil microarthropods. Pedobiologia.

[41] Snyder B.A., Boots B., Hendrix P.F. (2009). Competition between invasive earthworms (*Amynthas corticis*, Megascolecidae) and native North American millipedes (*Pseudopolydesmus erasus*, Polydesmidae): Effects on carbon cycling and soil structure. Soil Biol. Biochem..

[42] Joly F.-X., Coulis M., Gérard A., Fromin N., Hättenschwiler S. (2015). Litter-type specific microbial responses to the transformation of leaf litter into millipede feces. Soil Biol. Biochem..

[43] Brown S.P., Brogden M., Cortes C., Tucker A.E., VandeVoort A.R., Snyder B.A. (2021). Investigating the effects of nitrogen deposition and substrates on the microbiome and mycobiome of the millipede *Cherokia georgiana georgiana* (Diplopoda: Polydesmida). Soil Biol. Biochem..

[44] Golovatch S.I., Liu W. (2020). Diversity, distribution patterns, and fauno-genesis of the millipedes (*Diplopoda*) of mainland China. Zookeys.

[45] Gutiérrez López M., Isla García de Leaniz M., Trigo Aza D. (2020). Do cryptic species of earthworms affect soil arthropods differently? The case of the *Carpetania elisae* complex in the center of the Iberian Peninsula. Eur. J. Soil Biol..

[46] Cameron E.K., Knysh K.M., Proctor H.C., Bayne E.M. (2013). Influence of two exotic earthworm species with different foraging strategies on abundance and composition of boreal microarthropods. Soil Biol. Biochem..

[47] Zhu X., Chang L., Li J., Liu J., Feng L., Wu D. (2018). Interactions between earthworms and mesofauna affect CO_2_ and N_2_O emissions from soils under long-term conservation tillage. Geoderma.

[48] Gongalsky K.B. (2021). Soil macrofauna: Study problems and perspectives. Soil Biol. Biochem..

[49] Suzuki Y., Grayston S.J., Prescott C.E. (2013). Effects of leaf litter consumption by millipedes (*Harpaphe haydeniana*) on subsequent decomposition depends on litter type. Soil Biol. Biochem..

[50] Yin W.Y. (1998). Pictorial Keys to Soil Animals of China.

[51] Potapov M. (2001). Synopses on Palaearctic Collembola: Isotomidae.

[52] Balogh P., Balogh J. (2012). The Soil Mites of the World: Vol. 3: Oribatid Mites of the Neotropical Region II.

[53] Ryabinin N., Liu D., Gao M., WU D.-H. (2018). Checklist of oribatid mites (Acari, Oribatida) of the Russian Far East and Northeast of China. Zootaxa.

[54] Oksanen J., Blanchet F.G., Friendly M., Kindt R., Legendre P., McGlinn D., Minchin P.R., O’Hara R.B., Simpson G.L., Solymos P. (2019). Vegan: Community Ecology Package (p. R Package Version 2.5-4). https://CRAN.R-project.org/package=vegan.

[55] Kolde R., Kolde M.R. Package ‘pheatmap’. *R Package* 2015. R Package Version 1.0.12. https://cran.r-project.org/web/packages/pheatmap/pheatmap.pdf.

[56] Liu D., Liu D., Yu H., Wu H. (2023). Strong variations and shifting mechanisms of altitudal diversity and abundance patterns in soil oribatid mites (Acari: Oribatida) on the Changbai Mountain, China. Appl. Soil Ecol..

[57] Makoto K., Arai M., Kaneko N. (2014). Change the menu? Species-dependent feeding responses of millipedes to climate warming and the consequences for plant–soil nitrogen dynamics. Soil Biol. Biochem..

[58] Alvarez T., Frampton G.K., Goulson D. (2001). Epigeic Collembola in winter wheat under organic, integrated and conventional farm management regimes. Agr. Ecosyst. Environ..

[59] Wickenbrock L., Heisler C. (1997). Influence of earthworm activity on the abundance of Collembola in soil. Soil Biol. Biochem..

[60] Larsen T., Schjønning P., Axelsen J. (2004). The impact of soil compaction on euedaphic Collembola. Appl. Soil Ecol..

[61] Behan-Pelletier V.M. (1999). Oribatid mite biodiversity in agroecosystems: Role for bioindication. Agr. Ecosyst. Environ..

[62] Cameron E.K., Bayne E.M. (2011). An experimental test of facilitation between non-native earthworms. Can. J. Zool..

[63] Salmon S., Geoffroy J.-J., Ponge J.-F. (2005). Earthworms and collembola relationships: Effects of predatory centipedes and humus forms. Soil Biol. Biochem..

[64] Coulibaly S.F.M., Coudrain V., Hedde M., Brunet N., Mary B., Recous S., Chauvat M. (2017). Effect of different crop management practices on soil Collembola assemblages: A 4-year follow-up. Appl. Soil Ecol..

[65] Chamberlain S.A., Bronstein J.L., Rudgers J.A. (2014). How context dependent are species interactions?. Ecol. Lett..

[66] Song C., Von Ahn S., Rohr R.P., Saavedra S. (2020). Towards a probabilistic understanding about the context-dependency of species interactions. Trends Ecol. Evol..

[67] Hernandez D.J., David A.S., Menges E.S., Searcy C.A., Afkhami M.E. (2021). Environmental stress destabilizes microbial networks. ISME J.

[68] Lebrun P., van Straalen N.M. (1995). Oribatid mites: Prospects for their use in ecotoxicology. Exp. Appl. Acarol..

[69] Seniczak S., Graczyk R., Seniczak A., Faleńczyk-Koziróg K., Kaczmarek S., Marquardt T. (2018). Microhabitat preferences of Oribatida and mesostigmata (Acari) inhabiting lowland beech forest in Poland and the trophic interactions between these mites. Eur. J. Soil Biol..

[70] Maraun M., Scheu S. (2000). The structure of oribatid mite communities (Acari, Oribatida): Patterns, mechanisms and implications for future research. Ecography.

[71] Wallwork J.A. (1983). Oribatids in forest ecosystems. Annu. Rev. Entomol..

[72] Seniczak A., Seniczak S., Graczyk R., Kaczmarek S., Jordal B.H., Kowalski J., Djursvoll P., Roth S., Bolger T. (2021). A forest pool as a habitat island for mites in a limestone forest in Southern Norway. Diversity.

[73] Minor M.A., Cianciolo J.M. (2007). Diversity of soil mites (Acari: Oribatida, Mesostigmata) along a gradient of land use types in New York. Appl. Soil Ecol..

[74] Birkhofer K., Dietrich C., John K., Schorpp Q., Zaitsev A.S., Wolters V. (2016). Regional conditions and land-use alter the potential contribution of soil arthropods to ecosystem services in grasslands. Front. Ecol. Evol..

[75] Chauvat M., Zaitsev A.S., Wolters V. (2003). Successional changes of Collembola and soil microbiota during forest rotation. Oecologia.

[76] McLean M.A., Parkinson D. (2000). Introduction of the epigeic earthworm *Dendrobaena octaedra* changes the oribatid community and microarthropod abundances in a pine forest. Soil Biol. Biochem..

[77] Bonkowski M., Scheu S., Schaefer M. (1998). Interactions of earthworms (*Octolasion lacteum*), millipedes (*Glomeris marginata*) and plants (*Hordelymus europaeus*) in a beechwood on a basalt hill: Implications for litter decomposition and soil formation. Appl. Soil Ecol..

[78] Potapov A.M., Tiunov A.V. (2016). Stable isotope composition of mycophagous collembolans versus mycotrophic plants: Do soil invertebrates feed on mycorrhizal fungi?. Soil Biol. Biochem..

[79] Ponge J.-F. (2000). Vertical distribution of Collembola (Hexapoda) and their food resources in organic horizons of beech forests. Biol. Fertil. Soils..

[80] Chahartaghi M., Langel R., Scheu S., Ruess L. (2005). Feeding guilds in Collembola based on nitrogen stable isotope ratios. Soil Biol. Biochem..

[81] Hubert J., Žilová M., Pekár S. (2001). Feeding preferences and gut contents of three panphytophagous oribatid mites (Acari: Oribatida). Eur. J. Soil Biol..

[82] Meyer S., Kundel D., Birkhofer K., Fliessbach A., Scheu S. (2022). Trophic niche but not abundance of Collembola and Oribatida changes with drought and farming system. PeerJ.

[83] Siepel H., Ruiter-Dijkman E.M.d. (1993). Feeding guilds of oribatid mites based on their carbohydrase activities. Soil Biol. Biochem..

[84] Schneider K., Migge S., Norton R.A., Scheu S., Langel R., Reineking A., Maraun M. (2004). Trophic niche differentiation in soil microarthropods (Oribatida, Acari): Evidence from stable isotope ratios (^15^N/^14^N). Soil Biol. Biochem..

[85] Endlweber K., Ruess L., Scheu S. (2009). Collembola switch diet in presence of plant roots thereby functioning as herbivores. Soil Biol. Biochem..

[86] Huang Y., Yesilonis I., Szlavecz K. (2020). Soil microarthropod communities of urban green spaces in Baltimore, Maryland, USA. Urban For. Urban Green..

[87] Maraun M., Migge S., Schaefer M., Scheu S. (1998). Selection of microfungal food by six oribatid mite species (Oribatida, Acari) from two different beech forests. Pedobiologia.

