# Supplementary material for: Impacts of Millipedes on Acari and Collembola Communities—A Microcosm Experiment
Source: Insects. 2024 Jun 18;15(6):456. doi: 10.3390/insects15060456 (PMC11203639; doi:10.3390/insects15060456)
Supplement: Supplementary file 1 [file insects-15-00456-s001.zip › insects-2983540-supplementary.pdf]

Supplementary material

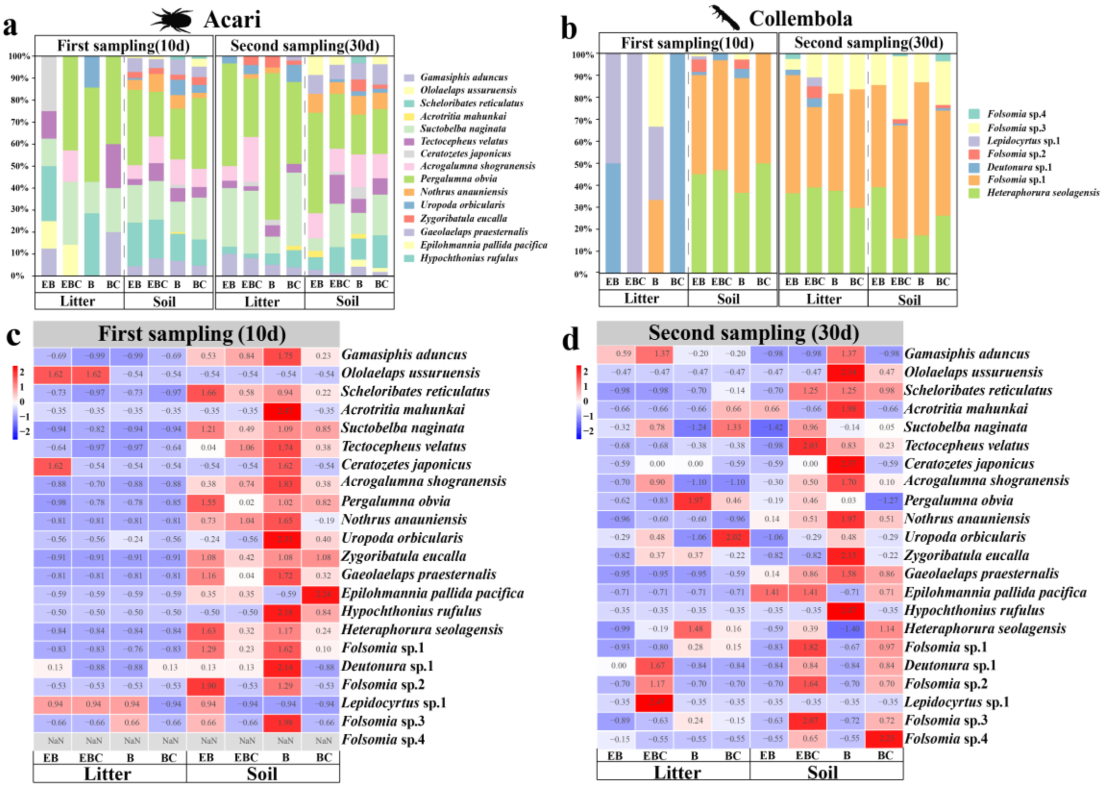

Figure S1. Stacked plots with percentage abundance of (a) Acari and (b) Collembola species in different treatments under two sampling sessions. Heatmap of correlation between different species under different treatments at first sampling (c) and second sampling (d).

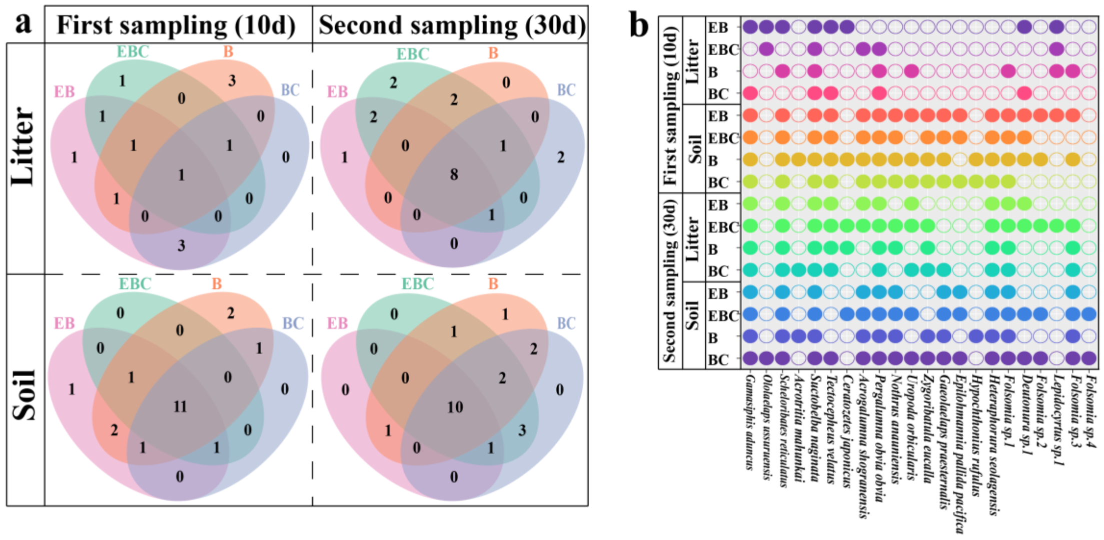

Figure S2. (a) Venn diagram of species in the litter and soil under two sampling sessions. (b) Bubble diagram of the distribution of different species under different treatments.

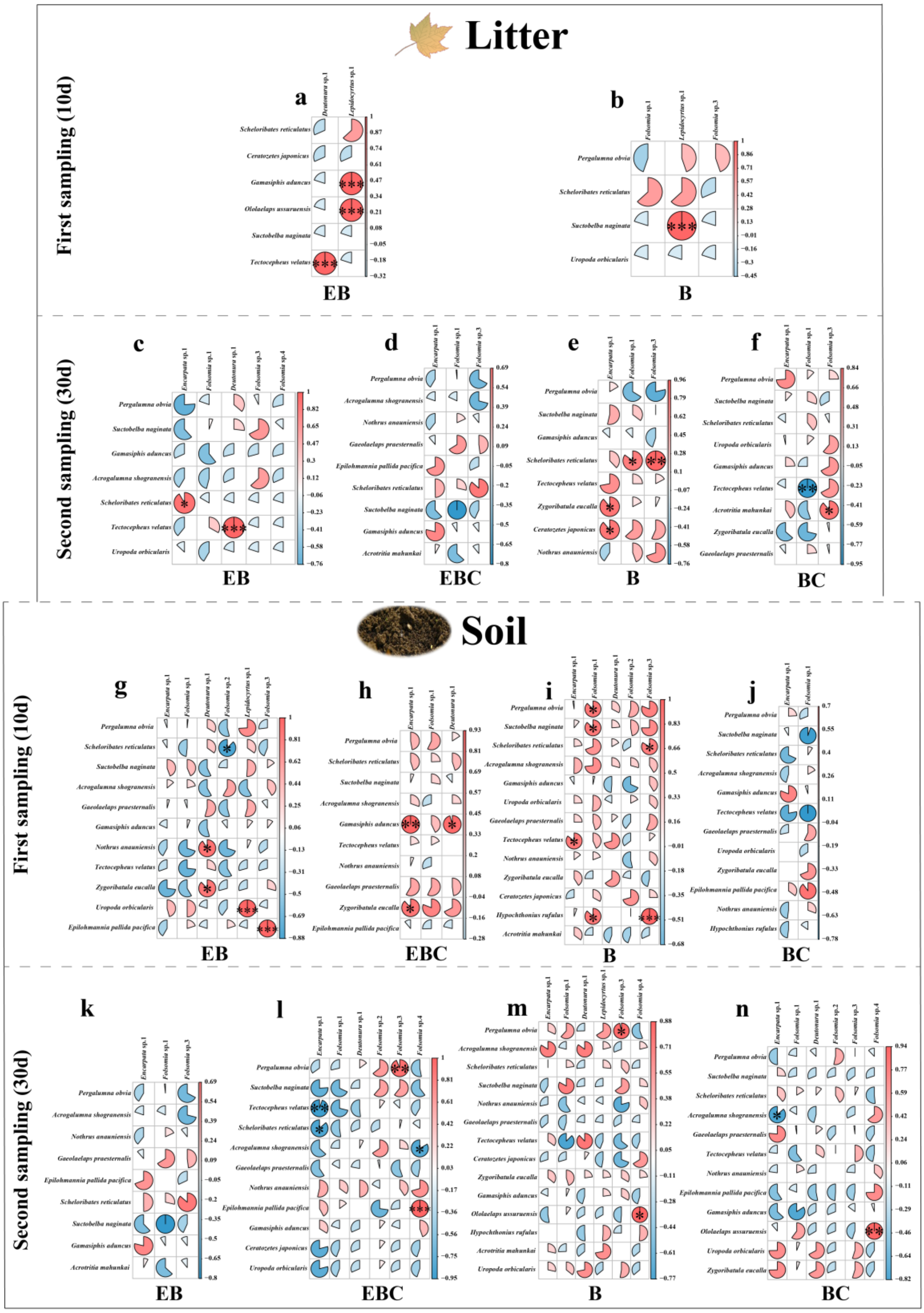

Figure S3. Heatmap of correlations between different species of acari and collembola under different treatments in the litter and soil. (Red is positive correlation and blue is negative correlation, the darker the color and the more complete the pie shape, the stronger the correlation. * $p < 0.05$; ** $p < 0.01$; *** $p < 0.001$)

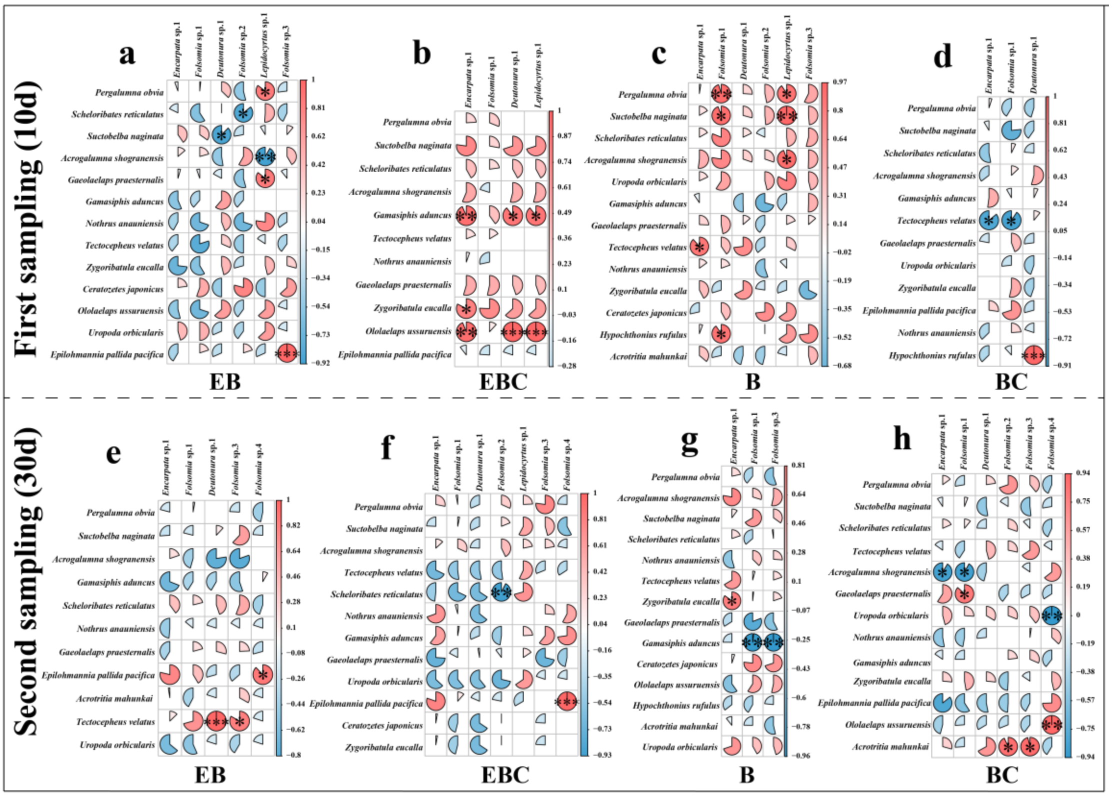

Figure S4. Heatmap of correlation between acari and collembola in different treatments. (Red is positive correlation and blue is negative correlation, the darker the color and the more complete the pie shape, the stronger the correlation.)

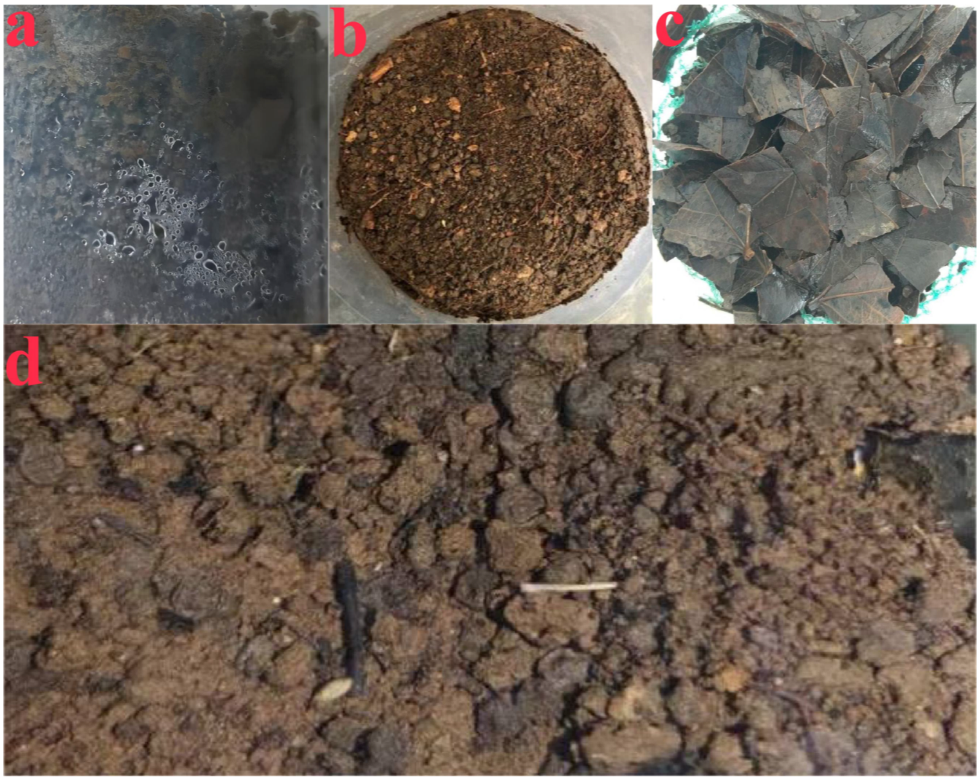

Figure S5. a. the pores produced by the millipede activity in the soil and it can also be observed that there is more water in the pores; b. the soil without millipede treatment, the soil as a whole is highly compacted without large pores; c. the feces left by the millipede in the litter layer and the breaking of the litter edge; d. the large amount of feces left by the millipede in the soil, all the round pie-shaped middle depressions in the figure are the feces produced by the millipede feces.

Table S1. Differences in Acari and Collembola abundance between treatments in the litter and soil at first sampling.

	**Litter - First sampling (10d)**					**Soil - First sampling (10d)**				
	**EB**	**EBC**	**B**	**BC**		**EB**	**EBC**	**B**	**BC**	
Gamasiphis aduncus	0.17±0.16	0	0	0.17±0.16	*p*>0.05	0.83±0.31	1.00±0.45	1.50±0.76	0.67±0.49	*p*>0.05
Ololaelaps ussuruensis	0.17±0.16	0.17±0.16	0a	0	*p*>0.05	0	0	0	0	——
Scheloribates reticulatus	0.33±0.21	0	0.33±0.21	0	*p*>0.05	3.67±0.76	2.17±0.87	2.67±0.88	1.67±0.56	*p*>0.05
Acrotritia mahunkai	0	0	0	0	——	0	0	0	0	——
Suctobelba naginata	0.17±0.16	0.33±0.33	0.17±0.16	0.17±0.16	*p*>0.05	3.17±1.11	2.17±0.31	3.00±0.63	2.67±0.92	*p*>0.05
Tectocepheus velatus	0.17±0.16	0	0	0.17±0.16	*p*>0.05	0.50±0.34	1.00±0.37	1.33±0.42	0.67±0.33	*p*>0.05
Ceratozetes japonicus	0.33±0.21a	0b	0b	0b	***p*<0.05**	0	0	0.33±0.21	0	*p*>0.05
Acrogalumna shogranensis	0	0.17±0.16	0	0	*p*>0.05	1.17±0.40	1.50±0.43	2.50±0.76	1.17±0.31	*p*>0.05
Pergalumna obvia	0	0.50±0.34	0.50±0.22	0.33±0.33	*p*>0.05	6.33±0.99a	2.50±0.67b	5.00±1.37ab	4.50±0.99ab	***p*<0.05**
Nothrus anauniensis	0	0	0	0	——	0.83±0.46	1.00±0.63	1.33±0.56a	0.33±0.33	*p*>0.05
Uropoda orbicularis	0	0	0.17±0.16	0	*p*>0.05	0.17±0.16b	0b	1.50±0.43a	0.50±0.22ab	***p*<0.01**
Zygoribatula eucalla	0	0	0	0	——	0.50±0.34	0.33±0.21	0.50±0.34	0.50±0.22	*p*>0.05
Gaeolaelaps praesternalis	0	0	0	0	——	1.17±0.31	0.50±0.22	1.50±0.56	0.67±0.49	*p*>0.05
Epilohmannia pallida pacifica	0	0	0	0	——	0.17±0.16	0.17±0.16	0	0.50±0.34	*p*>0.05
Hypochthonius rufulus	0	0	0	0	——	0	0	0.33±0.21	0.17±0.16	*p*>0.05
**Total Acari**	2.00±0.00	1.75±0.48	1.75±0.48	1.25±0.25	*p*>0.05	22.20±1.39ab	14.80±0.73b	26.00±1.95a	16.80±2.13ab	***p*<0.001**
Heteraphorura seolagensis	0	0	0	0	——	10.67±0.89a	5.00±1.00b	8.67±0.88a	4.67±0.33b	***p*<0.05**
Folsomia sp.1	0	0	0.17±0.16	0	*p*>0.05	5.33±0.67ab	2.67±0.56b	6.17±1.76a	2.33±0.56b	***p*<0.05**

Deutonura sp.1	0.17±0.16	0	0	0.17±0.16	$p>0.05$	0.17±0.16ab	0.17±0.16ab	0.50±0.22a	0b	**$p<0.05$**
Folsomia sp.2	0	0	0	0	——	0.67±0.33a	0b	0.50±0.22ab	0b	**$p<0.05$**
Lepidocyrtus sp.1	0.17±0.16	0.17±0.16	0.17±0.16	0	$p>0.05$	0.17±0.16	0	0	0	$p>0.05$
Folsomia sp.3	0	0	0.17±0.16	0	$p>0.05$	0.17±0.16	0	0.33±0.21	0	$p>0.05$
Folsomia sp.4	0	0	0	0	——	0	0	0	0	——
**Total Collembola**	0.67±0.33	0.33±0.33	1.00±0.00	0.33±0.33	$p>0.05$	23.67±2.33a	10.67±0.88b	23.67±3.38a	9.33±0.67b	**$p<0.01$**
**Total**	1.43±0.29	1.14±0.40	1.44±0.29	0.86±0.26	$p>0.05$	22.75±1.16a	13.25±0.92b	25.13±1.66a	14.000±1.88b	**$p<0.001$**

2

Table S2. Differences in Acari and Collembola abundance between treatments in the litter and soil at second sampling.

	**Litter - Second sampling (30d)**					**Soil - Second sampling (30d)**				
	**EB**	**EBC**	**B**	**BC**		**EB**	**EBC**	**B**	**BC**	
Gamasiphis aduncus	0.50±0.34	0.67±0.33	0.33±0.21	0.33±0.21	*p*>0.05	0.17±0.16	0.17±0.16	0.67±0.33	0.17±0.16	*p*>0.05
Ololaelaps ussuruensis	0	0	0	0	——	0	0	0.50±0.34	0.17±0.16	*p*>0.05
Scheloribates reticulatus	0.17±0.16	0.17±0.16	0.33±0.21	0.67±0.33	*p*>0.05	0.33±0.21	1.50±0.50	1.50±0.43	1.33±0.49	*p*>0.05
Acrotritia mahunkai	0	0	0	0.17±0.16	*p*>0.05	0.17±0.16	0	0.33±0.21	0	*p*>0.05
Suctobelba naginata	1.33±0.49ab	2.33±0.61a	0.50±0.22b	2.83±0.91a	***p*<0.05**	0.33±0.21b	2.50±0.62a	1.5±0.43ab	1.67±0.71ab	***p*<0.01**
Tectocepheus velatus	0.17±0.16	0.17±0.16	0.33±0.21	0.33±0.21	*p*>0.05	0b	1.67±0.71a	1.00±0.52ab	0.67±0.33ab	***p*<0.05**
Ceratozetes japonicus	0	0.17±0.16	0.17±0.16	0	*p*>0.05	0b	0.17±0.16b	0.83±0.40a	b	***p*<0.05**
Acrogalumna shogranensis	0.33±0.21ab	1.67±0.67a	0b	0b	***p*<0.01**	0.67±0.33b	1.33±0.71ab	2.33±0.71a	1.00±0.37ab	***p*<0.05**
Pergalumna obvia	2.33±0.92	2.17±0.31	4.33±1.92	3.17±0.75	*p*>0.05	2.67±0.92	3.17±0.70	2.83±0.75	1.83±0.87	*p*>0.05
Nothrus anauniensis	0	0.17±0.16	0.17±0.16	0	*p*>0.05	0.50±0.50	0.67±0.21	1.33±0.42	0.67±0.21	*p*>0.05
Uropoda orbicularis	0.17±0.16ab	0.33±0.21ab	0b	0.67±0.21a	***p*<0.05**	0	0.17±0.16	0.33±0.2	0.17±0.16	*p*>0.05
Zygoribatula eucalla	0	0.33±0.21	0.33±0.21	0.17±0.16	*p*>0.05	0b	0b	0.83±0.17a	0.17±0.16b	***p*<0.001**
Gaeolaelaps praesternalis	0	0	0	0.17±0.16	*p*>0.05	0.50±0.34	0.83±0.48	1.17±0.16	0.83±0.40	*p*>0.05
Epilohmannia pallida pacifica	0	0	0	0	——	0.50±0.34	0.50±0.22	0	0.33±0.21	*p*>0.05
Hypochthonius rufulus	0	0	0	0	——	0b	0b	0.50±0.22a	0b	***p*<0.01**
**Total Acari**	2.00±1.01	3.27±1.26	2.60±1.69	3.40±1.57	*p*>0.05	2.33±1.04b	5.07±1.54ab	6.27±1.19a	3.60±0.96ab	***p*<0.05**
Heteraphorura seolagensis	2.50±0.76b	4.83±1.35ab	9.67±2.88a	5.83±1.60ab	***p*<0.05**	3.67±1.05bc	6.50±0.88ab	1.33±0.21c	8.67±1.22a	***p*<0.01**
Folsomia sp.1	3.67±0.80b	4.50±1.23b	11.33±2.19a	10.50±1.33a	***p*<0.01**	4.33±0.92c	21.00±4.97a	5.33±1.174bc	15.67±1.17ab	***p*<0.01**

Deutonura sp.1	0.17±0.16	0.50±0.34	0	0	$p>0.05$	0	0.33±0.21	0	0.33±0.21	$p>0.05$
Folsomia sp.2	0b	0.67±0.33a	0b	0b	**$p<0.05$**	0b	0.83±0.31a	0ab	0.50±0.34ab	**$p<0.05$**
Lepidocyrtus sp.1	0b	0.55±0.22a	0b	0b	**$p<0.01$**	0	0	0	0	——
Folsomia sp.3	0.33±0.33b	1.33±0.71ab	4.67±1.58a	3.17±0.91ab	**$p<0.01$**	1.33±0.76b	11.67±1.69a	1.00±0.68b	6.50±2.13ab	**$p<0.001$**
Folsomia sp.4	0.17±0.16	0	0	0	$p>0.05$	0b	0.50±0.22ab	0b	1.17±0.60a	**$p<0.05$**
**Total Collembola**	8.20±3.43b	14.80±4.59ab	30.80±2.33a	23.40±1.96ab	**$p<0.001$**	18.67±5.46b	78.33±25.46a	15.33±8.35b	61.67±16.59ab	**$p<0.05$**
**Total**	10.00±2.64b	17.57±3.91ab	27.57±2.99a	24.00±1.45ab	**$p<0.001$**	18.20±1.20b	64.20±2.87a	28.00±4.04b	50.20±2.24a	**$p<0.001$**

Table S3. Differences in Acari and Collembola abundance in contrasting soils in the litter under different treatments at the first sampling.

First sampling (10d)	EB			EBC			B			BC		
	Litter	Soil		Litter	Soil		Litter	Soil		Litter	Soil	
Gamasiphis aduncus	0.17±0.16	0.83±0.31	$p>0.05$	0b	1.00±0.26a	**$p<0.05$**	0b	1.50±0.56a	**$p<0.05$**	0.17±0.16	0.67±0.49	$p>0.05$
Ololaelaps ussuruensis	0.17±0.16	0	$p>0.05$	0.17±0.16	0	$p>0.05$	0	0	——	0	0	——
Scheloribates reticulatus	0.33±0.21b	3.67±0.76a	**$p<0.01$**	0b	2.17±0.54a	**$p<0.01$**	0.33±0.21b	2.67±0.88a	**$p<0.05$**	0b	1.67±0.56a	**$p<0.05$**
Acrotritia mahunkai	0	0	——	0	0	——	0	0.17±0.16	$p>0.05$	0	0	——
Suctobelba naginata	0.17±0.16b	3.17±0.40a	**$p<0.001$**	0.33±0.33b	2.17±0.31a	**$p<0.01$**	0.17±0.16b	3.00±0.63a	**$p<0.01$**	0.17±0.16b	2.67±0.92a	**$p<0.05$**
Tectocepheus velatus	0.17±0.16	0.50±0.34	$p>0.05$	0b	1.00±0.37a	**$p<0.05$**	0b	1.33±0.42a	**$p<0.05$**	0.17±0.16	0.67±0.33	$p>0.05$
Ceratozetes japonicus	0.33±0.21	0	$p>0.05$	0	0	——	0	0.33±0.21	$p>0.05$	0	0	——
Acrogalumna shogranensis	0b	1.17±0.40a	**$p<0.05$**	0.17±0.16b	1.50±0.43a	**$p<0.05$**	0b	2.50±0.76a	**$p<0.05$**	0b	1.17±0.75a	**$p<0.05$**
Pergalumna obvia	0b	6.33±0.99a	**$p<0.001$**	0.50±0.34b	2.50±0.67a	**$p<0.05$**	0.50±0.22b	5.00±0.68a	**$p<0.001$**	0.33±0.33b	4.50±0.99a	**$p<0.01$**
Nothrus anauniensis	0	0.83±0.48	$p>0.05$	0	1.00±0.63	$p>0.05$	0b	1.33±0.42a	**$p<0.05$**	0	0.33±0.33	$p>0.05$
Uropoda orbicularis	0	0.17±0.16	$p>0.05$	0	0	——	0.17±0.16b	1.50±0.43a	**$p<0.05$**	0	0.50±0.22	$p>0.05$
Zygoribatula eucalla	0	0.50±0.34	$p>0.05$	0	0.33±0.21	$p>0.05$	0	0.50±0.34	$p>0.05$	0	0.50±0.22	$p>0.05$
Gaeolaelaps praesternalis	0b	1.17±0.31a	**$p<0.05$**	0	0.50±0.22	$p>0.05$	0b	1.50±0.56a	**$p<0.05$**	0	0.67±0.49	$p>0.05$
Epilohmannia pallida pacifica	0	0.17±0.16	$p>0.05$	0	0.17±0.16	$p>0.05$	0	0	——	0	0.50±0.34	$p>0.05$
Hypochthonius rufulus	0	0	——	0	0	——	0	0.33±0.21	$p>0.05$	0	0.17±0.16	$p>0.05$
**Total Acari**	0.53±0.16b	7.40±0.96a	**$p<0.001$**	0.47±0.24b	4.93±0.71a	**$p<0.001$**	0.47±0.24b	8.67±1.44a	**$p<0.001$**	0.33±0.16b	5.60±0.96a	**$p<0.001$**
Heteraphorura seolagensis	0b	5.33±1.61a	**$p<0.05$**	0b	2.50±0.96a	**$p<0.05$**	0b	4.33±0.95a	**$p<0.01$**	0b	2.33±0.71a	**$p<0.05$**
Folsomia sp.1	0b	5.33±0.67a	**$p<0.001$**	0b	2.67±0.56a	**$p<0.01$**	0.17±0.16b	6.17±1.33a	**$p<0.01$**	0b	2.33±0.56a	**$p<0.01$**
Deutonura sp.1	0.17±0.16	0.17±0.16	$p>0.05$	0	0.17±0.16	$p>0.05$	0	0.50±0.22	$p>0.05$	0.17±0.16	0	$p>0.05$
Folsomia sp.2	0	0.67±0.33	$p>0.05$	0	0	——	0	0.50±0.22	$p>0.05$	0	0	——
Lepidocyrtus sp.1	0.17±0.16	0.17±0.16	$p>0.05$	0.17±0.16	0	$p>0.05$	0.17±0.16	0	$p>0.05$	0	0	——
Folsomia sp.3	0	0.17±0.16	$p>0.05$	0	0	——	0.17±0.16	0.33±0.21	$p>0.05$	0	0	——
Folsomia sp.4	0	0	——	0	0	——	0	0	——	0	0	——
**Total Collembola**	0.29±0.18b	10.14±1.16a	**$p<0.001$**	0.14±0.14b	4.57±0.78a	**$p<0.001$**	0.42±0.20b	10.14±1.94a	**$p<0.01$**	0.14±0.14b	4.00±0.69a	**$p<0.001$**

**Total**	0.45±0.14b	8.27±0.98a	***p*<0.001**	0.36±0.17b	4.82±0.52a	***p*<0.001**	0.45±0.17b	9.14±0.89a	***p*<0.001**	0.27±0.12b	4.91±0.94a	***p*<0.001**

6

Table S4. Differences in Acari and Collembola abundance in contrasting soil in the litter under different treatments at the second sampling.

Second sampling (30d)	EB			EBC			B			BC		
	Litter	Soil		Litter	Soil		Litter	Soil		Litter	Soil	
Gamasiphis aduncus	0.50±0.34	0.17±0.16	$p>0.05$	0.67±0.33	0.17±0.16	$p>0.05$	0.33±0.21	0.67±0.33	$p>0.05$	0.33±0.21	0.17±0.16	$p>0.05$
Ololaelaps ussuruensis	0	0	——	0	0	——	0	0.50±0.34	$p>0.05$	0	0.17±0.16	$p>0.05$
Scheloribates reticulatus	0.17±0.16	0.33±0.21	$p>0.05$	0.17±0.16b	1.50±0.50a	**$p<0.05$**	0.33±0.21b	1.50±0.43a	**$p<0.05$**	0.67±0.21b	1.50±0.22a	**$p<0.05$**
Acrotritia mahunkai	0	0.17±0.16	$p>0.05$	0	0	——	0	0.33±0.21	$p>0.05$	0.17±0.16	0	$p>0.05$
Suctobelba naginata	1.33±0.49	0.33±0.21	$p>0.05$	2.33±0.61	2.50±0.62	$p>0.05$	0.60±0.24b	1.80±0.37a	**$p<0.05$**	2.83±0.31a	1.67±0.33b	**$p<0.05$**
Tectocepheus velatus	0.17±0.16	0	$p>0.05$	2.00±0.77a	0.20±0.20b	**$p<0.05$**	0.33±0.21	1.00±0.52	$p>0.05$	0.33±0.21	0.67±0.33	$p>0.05$
Ceratozetes japonicus	0	0	——	0.17±0.16	0.17±0.16	$p>0.05$	0.17±0.16	0.83±0.40	$p>0.05$	0	0	——
Acrogalumna shogranensis	0.33±0.21	0.67±0.33	$p>0.05$	1.67±0.67	1.33±0.71	$p>0.05$	0b	2.33±0.71a	**$p<0.01$**	0b	1.00±0.36a	**$p<0.05$**
Pergalumna obvia	2.33±0.91	2.67±0.91	$p>0.05$	2.17±0.31	3.16±0.70	$p>0.05$	4.33±1.92	2.83±0.75	$p>0.05$	3.17±0.75	1.83±0.87	$p>0.05$
Nothrus anauniensis	0	0.5±0.5	$p>0.05$	0.17±0.16	0.67±0.21	$p>0.05$	0.17±0.16b	1.33±0.42a	**$p<0.05$**	0b	0.67±0.21a	**$p<0.05$**
Uropoda orbicularis	0.17±0.16	0	$p>0.05$	0.33±0.21	0.17±0.16	$p>0.05$	0	0.33±0.21	$p>0.05$	0.67±0.21	0.17±0.16	$p>0.05$
Zygoribatula eucalla	0	0	——	0.33±0.21	0	$p>0.05$	0.33±0.21	0.83±0.17	$p>0.05$	0.17±0.16	0.17±0.16	$p>0.05$
Gaeolaelaps praesternalis	0	0.50±0.34	$p>0.05$	0b	0.83±0.48a	**$p<0.05$**	0b	1.16±0.17a	**$p<0.001$**	0.17±0.16	0.83±0.40	$p>0.05$
Epilohmannia pallida pacifica	0	0.50±0.34	$p>0.05$	0b	0.50±0.22a	**$p<0.05$**	0	0	——	0	0.33±0.21	$p>0.05$
Hypochthonius rufulus	0	0	——	0	0	——	0b	0.50±0.22a	**$p<0.05$**	0	0	——
**Total Acari**	0.33±0.09	0.39±0.10	$p>0.05$	1.32±0.20b	2.05±0.23a	**$p<0.05$**	0.71±0.27b	1.71±0.14a	**$p<0.001$**	1.02±0.22	1.08±0.17	$p>0.05$
Heteraphorura seolagensis	2.50±1.87	3.67±1.05	$p>0.05$	4.83±1.35	6.50±0.88	$p>0.05$	9.67±1.05a	1.33±0.21b	**$p<0.001$**	5.83±0.91b	8.67±0.42a	**$p<0.05$**
Folsomia sp.1	3.67±0.80	4.33±0.91	$p>0.05$	4.50±1.23b	21.00±4.98a	**$p<0.01$**	11.33±1.05a	5.33±0.56b	**$p<0.001$**	10.50±1.33b	15.67±1.17a	**$p<0.05$**
Deutonura sp.1	0.17±0.16	0	$p>0.05$	0.50±0.34	0.33±0.21	$p>0.05$	0	0	——	0	0.33±0.21	$p>0.05$
Folsomia sp.2	0	0	——	0.67±0.33	0.83±0.31	$p>0.05$	0	0	——	0	0.50±0.34	$p>0.05$
Lepidocyrtus sp.1	0	0	——	0.50±0.22	0	$p>0.05$	0	0	——	0	0	——
Folsomia sp.3	0.33±0.33	1.33±0.76	$p>0.05$	1.33±0.71b	11.67±1.69a	**$p<0.001$**	4.67±0.76a	1.00±0.26b	**$p<0.01$**	3.17±0.17b	6.50±1.15a	**$p<0.05$**
Folsomia sp.4	0.17±0.16	0	$p>0.05$	0	0.50±0.22	$p>0.05$	0	0	——	0b	1.17±0.17a	**$p<0.001$**
**Total Collembola**	0.98±0.26	1.33±0.34	$p>0.05$	1.76±0.39b	5.83±1.35a	**$p<0.01$**	8.56±1.42a	2.56±0.64b	**$p<0.001$**	4.50±0.48b	7.58±0.77a	**$p<0.001$**

**Total**	2.02±0.29	2.60±0.35	$p>0.05$	0.93±0.15b	2.43±0.48a	**$p<0.05$**	2.78±0.30a	2.03±0.16b	**$p<0.05$**	2.80±0.29b	4.18±0.52a	**$p<0.05$**

8
